# How experimental trial context affects perceptual categorization

**DOI:** 10.3389/fpsyg.2015.00180

**Published:** 2015-02-19

**Authors:** Thomas J. Palmeri, Michael L. Mack

**Affiliations:** ^1^Department of Psychology, Vanderbilt Vision Research Center, Vanderbilt UniversityNashville, TN, USA; ^2^Center for Learning and Memory, The University of TexasAustin, TX, USA

**Keywords:** categorization, category learning, context, basic-level, experimental design

## Abstract

To understand object categorization, participants are tested in experiments often quite different from how people experience object categories in the real world. Learning and knowledge of categories is measured in discrete experimental trials, those trials may or may not provide feedback, trials appear one after another, after some fixed inter-trial interval, with hundreds of trials in a row, within experimental blocks with some structure dictated by the experimental design. In the real world, outside of certain educational and vocational contexts, opportunities to learn and use categories are intermixed over time with a whole multitude of intervening experiences. It is clear from any elementary understanding of human cognition that sequential effects matter, yet this understanding is often ignored, and categorization trials are often instead treated as independent events, immune to local trial context. In this perspective, we use some of our work to illustrate some of the consequences of the fact that categorization experiments have a particular trial structure. Experimental trial context can affect performance in category learning and categorization experiments in ways that can profoundly affect theoretical conclusions.

## INTRODUCTION

We need to be able to recognize objects in the world as *kinds of things* because we rarely see the exact same object twice. Kinds of things are called *categories*. On a hike in the woods, if we see a large brown creature, we need to know whether we should start slowly backing away or pull out our camera and snap a picture. If we see a tasty-looking mushroom at the base of a tree, we need to know if eating it might send us to the hospital. If we see a person walking toward us along the trail, we need to know if it is a friend or a stranger. These decisions all require categories.

On our hike, in one moment we might categorize the small creature in the tree before us as an *Indigo bunting*, then in the next moment notice that the tree itself is some kind of oak, not knowing the particular variety. Our friend suggests that it might be a *Black oak*. We then see a disgusting black insect with a vicious “tail” on a leaf in some foliage at the base of the tree. The park ranger leading the hike tells us that this is a kind of *Ichneumon wasp*, and thankfully notes that its extremely long ovipositor is not used for stinging but for laying its eggs in rotten trees. Telling birds from trees from bushes from bugs is categorization. Telling apart our friend from the park ranger is categorization. In a few moments of experience, many different categorizations of different objects at different levels of abstraction happen, with opportunities to learn a new kind of tree and a new kind of insect thrown in.

Laboratory experiments are far more structured. Consider a fairly typical category learning experiment (Richler and Palmeri, 2014). A small collection of novel objects – they could be random dot patterns (Posner and Keele, 1968), novel shapes (Op de Beeck et al., 2001), cartoon animals (Folstein et al., 2010), and so forth – are created and participants must learn what category each object belongs in. On every category learning trial, a single object is presented, the participant responds with a category label, corrective feedback is provided, and then after some brief delay, the next trial begins. Sometimes participants are tested on old trained objects and new transfer objects without feedback, sometimes at the end of learning and sometimes over the course of learning (Johansen and Palmeri, 2002). In nearly every category learning experiment, participants are tested on a collection of objects from a small number of categories, trial after trial after trial for 100s, sometimes 1000s, of trials at a stretch. While there may be certain educational and vocational contexts where people repeatedly perform the same categorizations on the same set of objects repeatedly, over and over again, this is clearly not like our hike in the woods.

Are there consequences of doing the same categorizations from one trial to the next in an experiment? Sometimes work aims precisely to understand sequential effects (e.g., Nelson et al., 2010), perhaps to investigate constructs like priming (e.g., McNamara, 2004) or task switching (e.g., Monsell, 2003). And important work in categorization aims to explore the role of sequential effects (e.g., Jones and Sieck, 2003; Jones et al., 2006) and how explicit variation to trial context can influence how categories are learned (e.g., Elio and Anderson, 1984; Mathy and Feldman, 2009; Pashler and Mozer, 2013; Carvalho and Goldstone, 2014a,b). But outside of work explicitly aimed at measuring or manipulating trial context, sequential effects, and the particulars of experimental trial structure are often ignored completely. Or they are acknowledged grudgingly, perhaps in the same vein as things like general fatigue effects, something that surely affects performance in experiments, but in theoretically uninteresting ways. Now, whereas constructs like sequential effects, priming, and task switching are defined as changes that occur from one trial to the next, a construct like categorization must exist outside of a particular trial context. By this we mean that while categorization can certainly be influenced by local context, some aspect of our ability to categorize object should be independent of that context. So we need to recognize when some of the effects we observe about categorization are sensitive to trial context, especially since in the real world, particular categorizations need not be performed one after another, like they typically are in an experiment.

In this brief perspective, we illustrate previously unrecognized effects of trial context during category learning of novel objects, during category transfer after learning novel objects, and during categorization of known objects. In each case, a focus on particular experimental design details, particularly related to experimental trial context, will prove critical for a theoretical understanding of categorization (see also Richler and Palmeri, 2014). We focus here on some of our own work, but reference related work from other groups. In contrast to work that explicitly measures sequential effects or manipulates trial context, here examine the theoretical consequence of ignoring those effects entirely.

## CATEGORIZATION BY RELATIVE JUDGMENT

Consider the simple case of 10 objects that vary along a single dimension psychological continuum (e.g., size, brightness, or frequency):


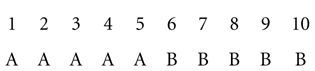


The first five objects are assigned to category A, the second five to category B (adapted from Stewart et al., 2002). Imagine we are partway through a category learning experiment. A participant is shown object 3 and asked what category it belongs to. Most theories of categorization would say that the object is compared to some internal representation of learned category knowledge that is stored in long-term memory (Palmeri and Tarr, 2008), which could be something like prototypes (e.g., Smith and Minda, 1998), exemplars (e.g., Nosofsky, 1986), or a decision rule (e.g., Ashby and Gott, 1988).

An alternative strategy does not rely on long-term memory at all, but on a relative judgment strategy based on recently experienced information in working memory, perhaps just the previous category learning trial in fact. On the trial right before object 3 was shown, the participant was shown some other object. Suppose it was object 10. They responded with category A or category B, and were then given feedback that object 10 is a member of category B. Instead of relying on a long-term category representation to categorize object 3, the participant could simply note that object 3 is very different from object 10, so it likely belongs in the other category, and responds, correctly, that it is a member of category A. Now if the previous object was instead object 2 and the participant was told that it belongs in category A, well, object 3 is very similar to object 2, so perhaps it also belongs to category A, which it does. For objects far from the category boundary, this relative judgment strategy will work quite well, whereas for objects close to the category boundary, it will work quite poorly, but that predicted performance maps well onto the performance you might see in an experiment. Although this strategy will not capture asymptotic levels of performance, when participants are perfect at categorizing all objects along the continuum, this strategy does predict fairly clear sequential effects, which in fact are observed in categorization (Stewart et al., 2002; Stewart and Brown, 2004; see also Carvalho and Goldstone, 2014b). And a variant of this relative judgment strategy can also account for performance in absolute identification (Stewart et al., 2005; but see Brown et al., 2007).

Are there broader theoretical consequences of participants using a simple relative judgment strategy of comparing the current object with the previous object and using its feedback? Imagine that, unbeknownst to the experimenter, this is precisely the strategy that participants use, without creating any long-term memory representations of categories. But imagine that the experimenter only considers that participant performance is based on forming long-term representations. Palmeri and Flanery (2001) simulated performance of participants using the simple relative judgment strategy for a sample of category structures (from Smith et al., 1997; Smith and Minda, 1998). They first asked whether the strategy was viable; would simulated participants perform greater than chance. They next asked whether that performance, which depends on only on a simple relative judgment strategy, would look more like performance predicted by a prototype abstraction model or an exemplar storage model, which both depend on long-term memory representations.

Rather than train people on objects that varied along a single dimension, Smith et al. (1997) trained people on objects that varied along multiple binary-valued dimensions. To illustrate, imagine that each category instance was defined by four dimensions that could each take on one of two values, designated abstractly as 0 for one value and 1 for the other value along a dimension. And imagine that category A members are 0 0 0 0, 0 0 0 1, 0 0 1 0, 0 1 0 0, and 1 0 0 0 and that category B members are the complement, 1 1 1 1, 1 1 1 0, 1 1 0 1, 1 0 1 1, and 0 1 1 1. At some point during learning, if you were to see item 1 1 1 0 and if the previous item was 0 0 1 0, labeled category A, it would be trivial to guess that 1 1 1 0 was in category B.

Palmeri and Flanery (2001) simulated predicted categorization behavior using this simple strategy over 14 different category learning problems reported by Smith et al. (1997) and Smith and Minda (1998). Not every category learning problem studied by Smith, or by others, can utilize this simple strategy; some kind of true long-term category learning is required in those cases. But for every category learning problem where Palmeri and Flanery (2001) reported that the simple strategy produced above chance categorization performance, Smith et al. (1997) had reported evidence for prototype abstraction over exemplar storage. Moreover, category learning performance that Palmeri and Flanery (2001) simulated using the simple strategy was better fitted by a prototype model than an exemplar model. Of course, performance was not based on prototypes in any of these cases, it was based only on memory for the previous trial. Behavior that seems to indicate the creation of particular kinds of long-term category representations can instead reflect strategies that are only possible because category learning experiments have a particular experimental trial context.

## CATEGORY LEARNING DURING CATEGORY TESTING

Our next example also comes from theoretical work contrasting exemplars and prototypes, specifically work that associates exemplars with an explicit declarative memory system and prototype abstraction with an implicit learning system (Squire and Zola, 1996). One well-known demonstration of a behavioral dissociation between the two, tested amnesic individuals and controls on category learning and recognition memory with a variant of the classic dot pattern task (Knowlton and Squire, 1993; Squire and Knowlton, 1995). After studying a small set of random dot patterns (Posner and Keele, 1968), amnesic individuals were significantly impaired compared to controls at discriminating studied dot patterns from new dot patterns in a recognition memory test. However, after studying a set of distortions of a prototype dot pattern, there was no significant difference between amnesic individuals and controls in their ability to categorize members versus non-members at test. By itself, a significantly larger impairment at recognition memory compared to categorization is not necessarily inconsistent with predictions of exemplar models (Nosofsky and Zaki, 1998). However, observing chance recognition memory with above chance categorization (Squire and Knowlton, 1995) is more theoretically challenging (Palmeri and Flanery, 2002).

But, here again is a case where it is important to closely examine the experimental trial context. For the recognition memory task, individuals studied five entirely random dot patterns that were presented eight times each. Then, they were tested on those five studied patterns and five new random patterns. Without memory for studied dot patterns, there is simply no way to discriminate old from new patterns. For the categorization task, individuals studied forty high-level distortions of a single category prototype. At test, they were shown four repetitions of the previously unseen prototype, 20 low-level distortions, 20 high-level distortions, and 40 completely new random patterns, and were asked to discriminate studied category members from non-members. While no feedback was provided in the categorization test, it is important to note that during the test subjects were shown many dot patterns that were very similar to one another and other dot patterns that were completely dissimilar from one another. Are long-term category representations even needed to discriminate members from non-members at test given the highly structured nature of the test itself?

Palmeri and Flanery (1999, 2002) conducted experiments where subjects were told that they had been subliminally shown a series of dot patterns during an initial unrelated task at the start of the experimental session. In fact, they had never seen any dot patterns whatsoever. Nothing was presented subliminally. Subjects were then tested on either the recognition memory or categorization dot pattern task. Not surprisingly, subjects were completely at chance at recognizing “old” dot patterns from new dot patterns; without memory (since no dot patterns had ever been seen before) there was simply no way to make that discrimination. However, even without ever having seen any previous distortions of the prototype, subjects were significantly above chance at categorization. In fact, these subjects who studied nothing performed just as well as subjects who had actually studied dot patterns earlier. Subjects had learned during the test, and Palmeri and Flanery (1999, 2001) surmised that this learning could be supported by working memory for a few recent testing trials, which is spared in amnesia. The potential for learning during test has since been shown to play a key role in several other experiments purporting to show dissociations between categorization and recognition memory (Zaki and Nosofsky, 2001, 2004, 2007; Nosofsky et al., 2012). Seemingly inconsequential details of the experimental trial context can have profound theoretical consequences.

## HOW BLOCK STRUCTURE AFFECTS CATEGORIZATION

Our final example switches gears to examine categorization performance with known categories rather than novel category learning. Most objects are categorized at an intermediate level of abstraction (e.g., *bird*) than more superordinate levels (e.g., *animal*) or subordinate levels (e.g., *Indigo bunting*). The so-called basic level advantage (Rosch et al., 1976) has been a cornerstone finding of significant work in categorization for many years (Mack and Palmeri, 2011). One place where the basic-level advantage is observed is the widely-used category verification task, where on each trial a category label is shown – on some trials it might be the subordinate level (*Indigo bunting*), on other trials the basic level (*bird*), and on other trials the superordinate level (*animal*) – then an object is shown and participants must verify whether the objects is a member of the target category or not as quickly and accurately as possible. For novice categories, basic-level category verifications are made significantly faster than subordinate or superordinate categorizations (cf., Tanaka and Taylor, 1991).

The basic-level advantage is likely familiar to anyone who has taken an introductory cognitive psychology class as an undergraduate. But, a different story emerges when the test image is shown with limited exposure duration. During an ultra-rapid categorization task, when images are shown for 30 ms or less, superordinate categorization is observed to be significantly faster than basic or subordinate categorization (Thorpe et al., 1996; Fabrè-Thorpe, 2011). Whether the basic level or superordinate level is prior, and why, has important theoretical implications. For Rosch et al. (1976) the basic-level carves nature at its joints. While for Thorpe et al. (1996) the superordinate level represents the initial feed-forward sweep through the visual system.

Most contrasts between speeded category verification (á la Rosch et al., 1976) and ultra-rapid categorization (á la Thorpe et al., 1996) have largely focused on the salient difference in exposure duration. But, with closer examination, it becomes clear that these tasks differ as well in their experimental trial context. Ultra-rapid categorization tasks block category context, testing participants on a single categorization at a single level of abstraction for hundreds of trials. Speeded category verification tasks, by contrast, mix categorizations at different levels of abstraction from trial to trial. When comparing across the two kinds of tasks, exposure duration and category context are confounded. Mack and Palmeri (under review) broke this confound, systematically investigating how both factors, exposure duration and category context, affect the relative speed of categorizations at different levels of abstraction. Of course, one possibility is that deconfounding exposure duration and category context might reveal that only exposure duration affected categorization. But that is not what we observed.

The basic-level advantage was eliminated only when stimulus exposure duration was brief and target category context was blocked. When exposure duration was brief and target category context was mixed, with categorization at different levels of abstraction from trial to trial, the ubiquitous basic-level advantage was preserved. Furthermore, within a mixed block of trials, when categorizations at a given level of abstraction happened to repeat over several trials, the basic-level advantage gave way to a growing superordinate advantage. This novel finding presents a challenge to extant theories, and space precludes a complete discussion (see Mack and Palmeri, under review). What is clear is that whether or not there is observed a superordinate advantage with brief exposure seems to depend critically on experimental trial context. Experimenters ignoring trial context do so at their peril.

## SUMMARY AND DISCUSSION

The only thing worse than a language maven is a statistics maven – and the only thing worse than a statistics maven is a methods maven. Every experimental psychologist knows it is folly to treat experimental trials as independent from one another. Sequential effects are ever-present, and may be key to theoretical understanding (e.g., Jones et al., 2013; Jones, 2014). Here we have roused the methods maven to highlight how experimental trial context can profoundly influence category learning and categorization behavior. These are not simply quantitative differences, but qualitative influences with potentially profound theoretical implications. Oddly, while significant work cited earlier has demonstrated sequential effects or has manipulated trial context, work not focused on measuring or manipulating trial context has too often implicitly assumed no systematic influence of trial context on categorization.

In the introduction, we highlighted how categorization in the real world often jumps from one kind of object to another kind of object, from one level of abstraction to another level of abstraction. Our choice of reference to the real world was not intended as a screed on ecological validity – like Mook (1983), we will defend external invalidity to our dying day. Instead it was to highlight that, unlike a domain like task switching, where the real world analog is just that, switching from one task to another, in categorization, sometimes we follow up one categorization with another of the same sort, but often we do not. Our experiments and theories need to explain one-shot categorization. And to the extent that categorization behavior is sensitive to experimental trial context, we need to know when, how, and why.

## Conflict of Interest Statement

The authors declare that the research was conducted in the absence of any commercial or financial relationships that could be construed as a potential conflict of interest.
